# Supplementary figures and images for: Structural mechanism of strand exchange by the RAD51 filament
Source: eLife. 2025 Aug 18;14:RP107114. doi: 10.7554/eLife.107114 (PMC12360782; doi:10.7554/eLife.107114)

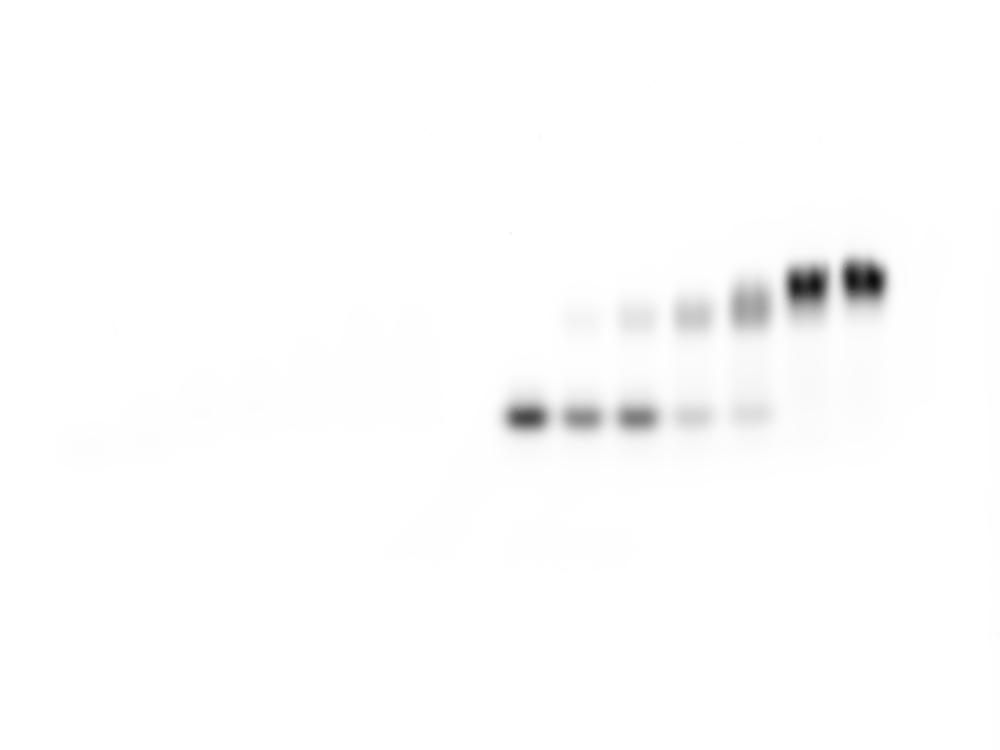

Supplement: Figure 1—figure supplement 1—source data 1. [file elife-107114-fig1-figsupp1-data1.zip › Figure 1-figure supplement 1-source data 1/Figure 1-figure supplement 1B [Cy3].tif]

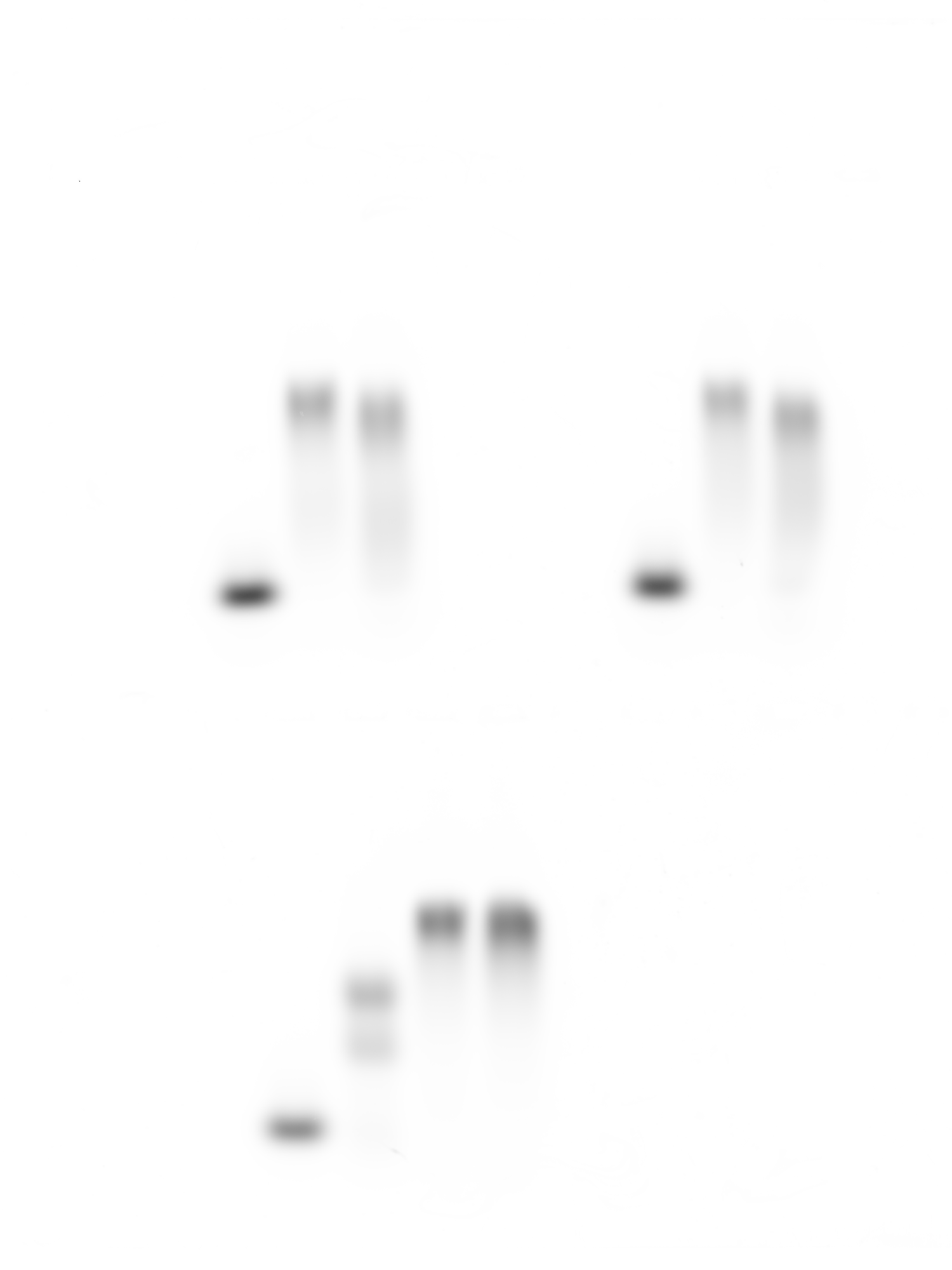

Supplement: Figure 1—figure supplement 1—source data 1. [file elife-107114-fig1-figsupp1-data1.zip › Figure 1-figure supplement 1-source data 1/Figure 1-figure supplement 1C [Cy3].tif]

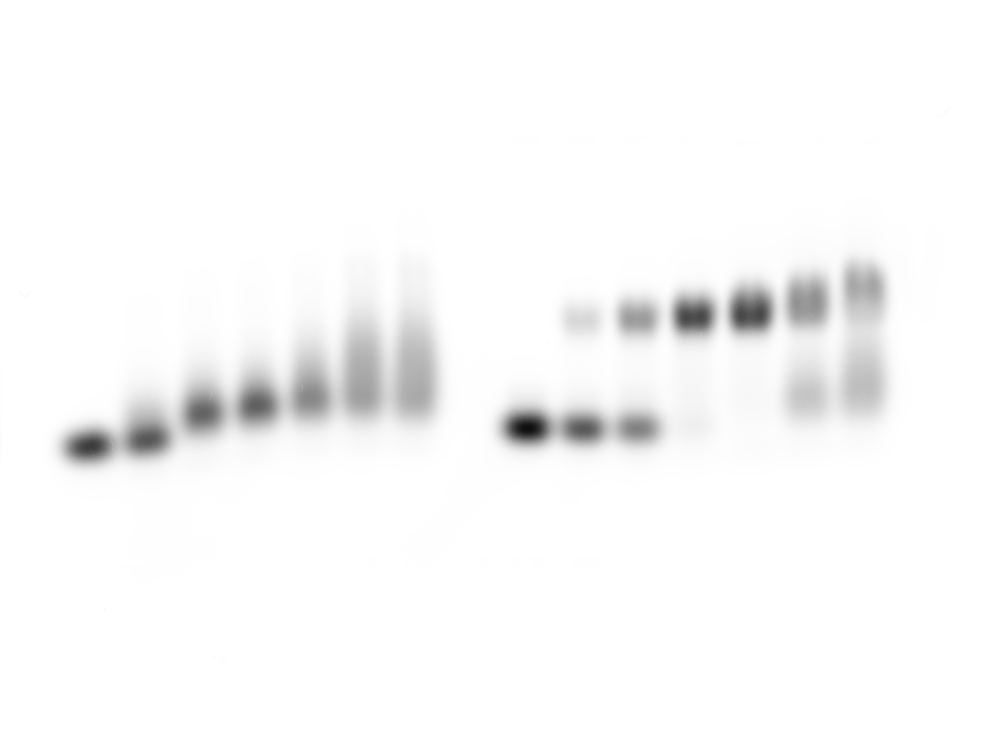

Supplement: Figure 1—figure supplement 1—source data 1. [file elife-107114-fig1-figsupp1-data1.zip › Figure 1-figure supplement 1-source data 1/Figure 1-figure supplement 1B [Cy5].tif]

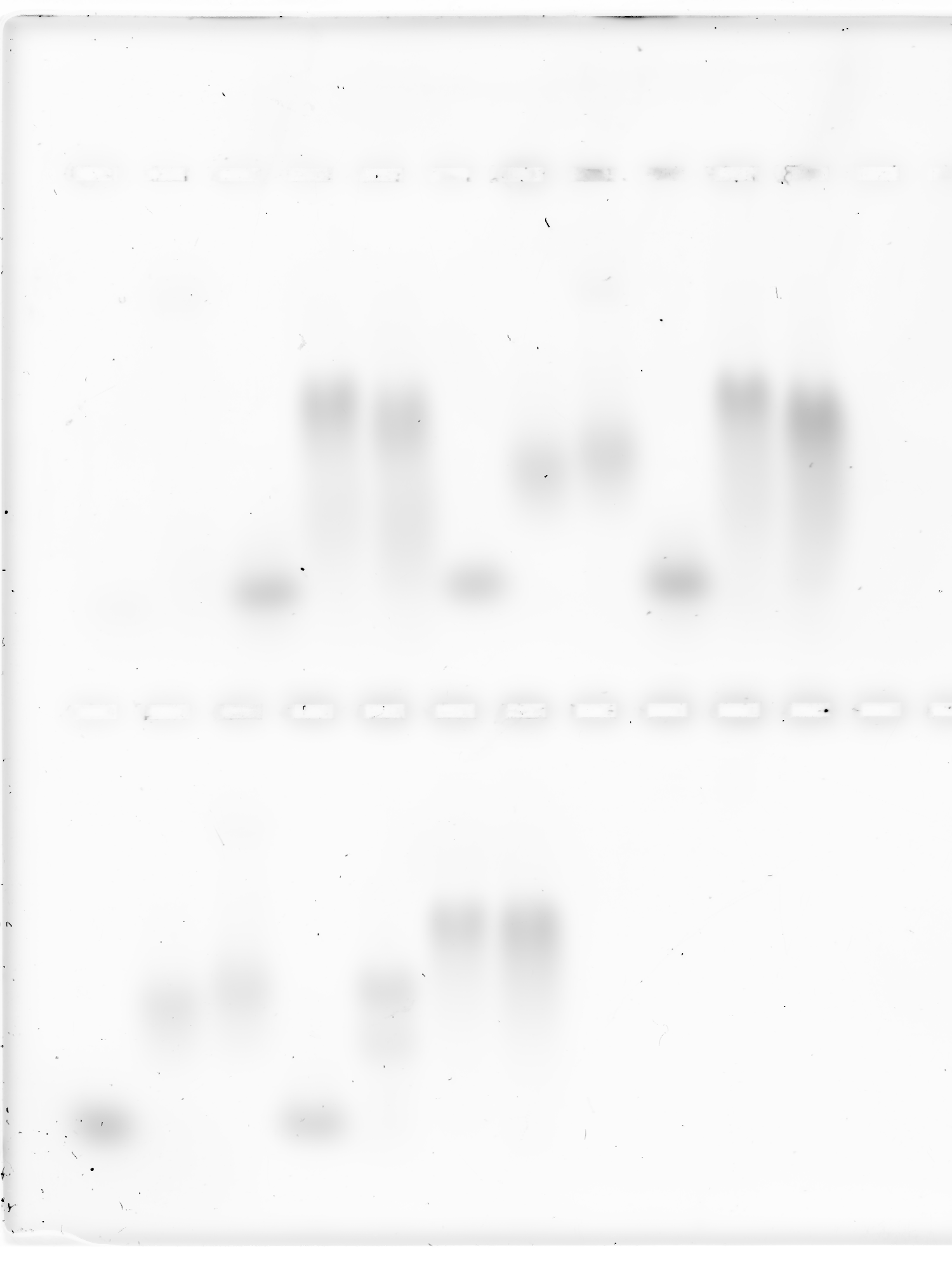

Supplement: Figure 1—figure supplement 1—source data 1. [file elife-107114-fig1-figsupp1-data1.zip › Figure 1-figure supplement 1-source data 1/Figure 1-figure supplement 1C [SYBR Gold].tif]

|                     | 12 | 13 | 14 | 15 | 16 | 17 | 18 |
|---------------------|----|----|----|----|----|----|----|
| ssDNA (0.5 $\mu$ M) | +  | +  | +  | -  | -  | -  | +  |
| dsDNA (0.5 $\mu$ M) | -  | -  | -  | +  | +  | +  | +  |
| mSA (5 $\mu$ M)     | -  | +  | +  | -  | +  | +  | +  |
| RAD51 (10 $\mu$ M)  | -  | -  | +  | -  | -  | +  | +  |

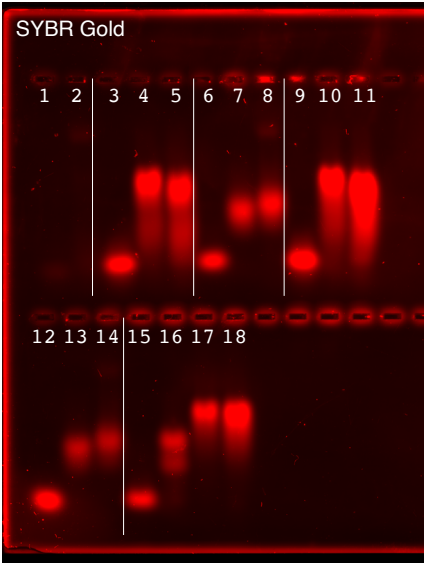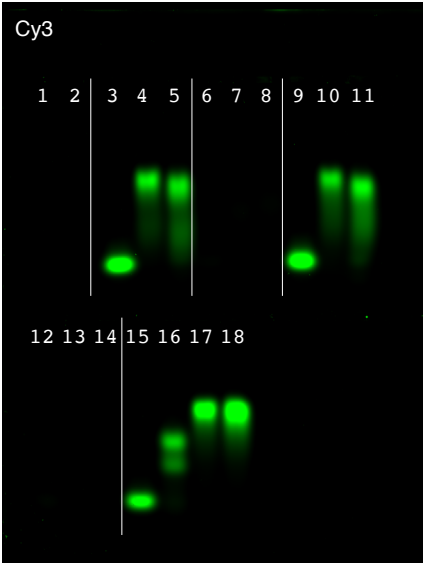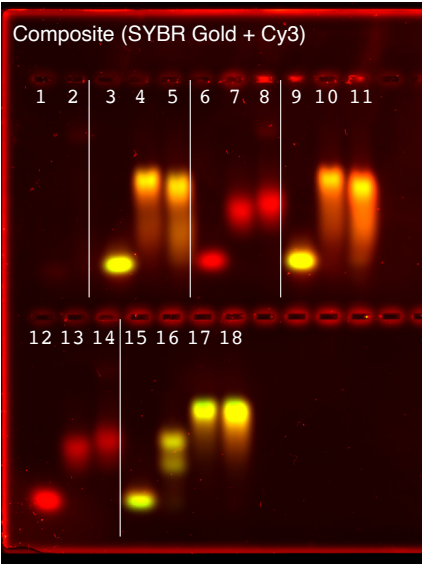

Supplement: Figure 1—figure supplement 1—source data 2. [file elife-107114-fig1-figsupp1-data2.zip › Figure 1-figure supplement 1-source data 2/Figure 1-figure supplement 1C GEL.pdf]

|                     |   |   |     |   |    |    |    |   |   |     |   |    |    |    |
|---------------------|---|---|-----|---|----|----|----|---|---|-----|---|----|----|----|
| ssDNA (0.5 $\mu$ M) | + | + | +   | + | +  | +  | +  | + | + | +   | + | +  | +  | +  |
| dsDNA (0.5 $\mu$ M) | - | - | -   | - | -  | -  | -  | + | + | +   | + | +  | +  | +  |
| RAD51 ( $\mu$ M)    | 0 | 1 | 2.5 | 5 | 10 | 20 | 30 | 0 | 1 | 2.5 | 5 | 10 | 20 | 30 |

Cy3

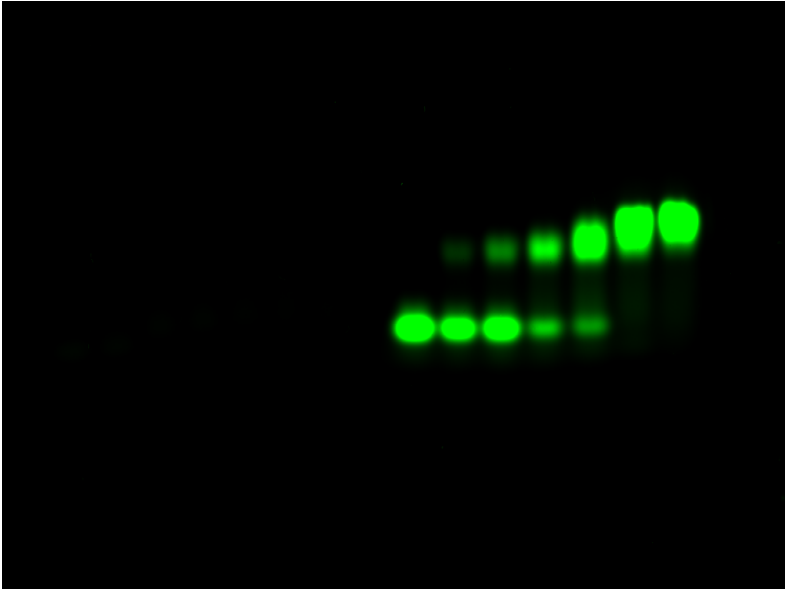

Cy5

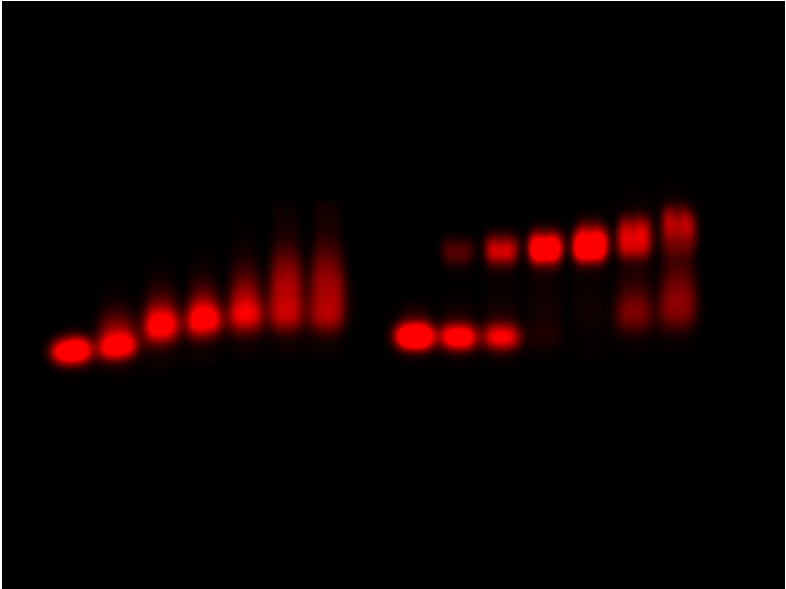

Merge

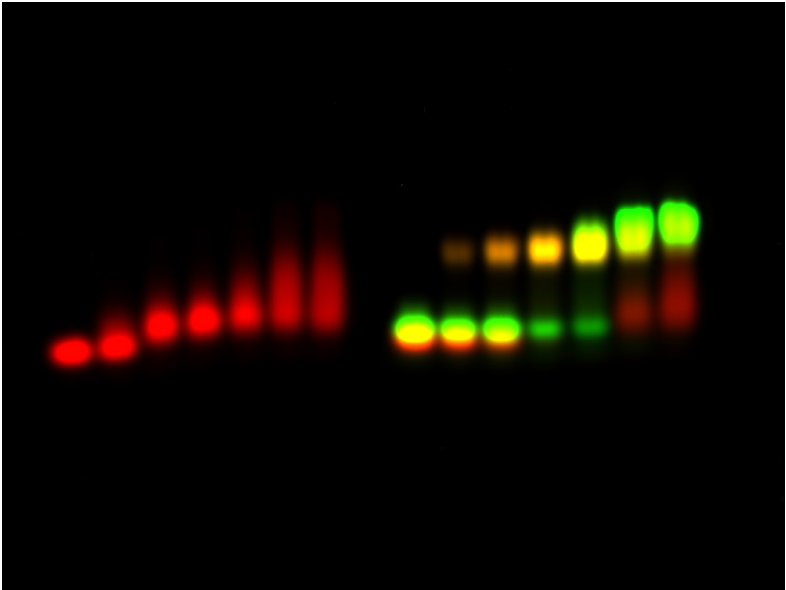

Supplement: Figure 1—figure supplement 1—source data 2. [file elife-107114-fig1-figsupp1-data2.zip › Figure 1-figure supplement 1-source data 2/Figure 1-figure supplement 1B-source data 1 GEL.pdf]

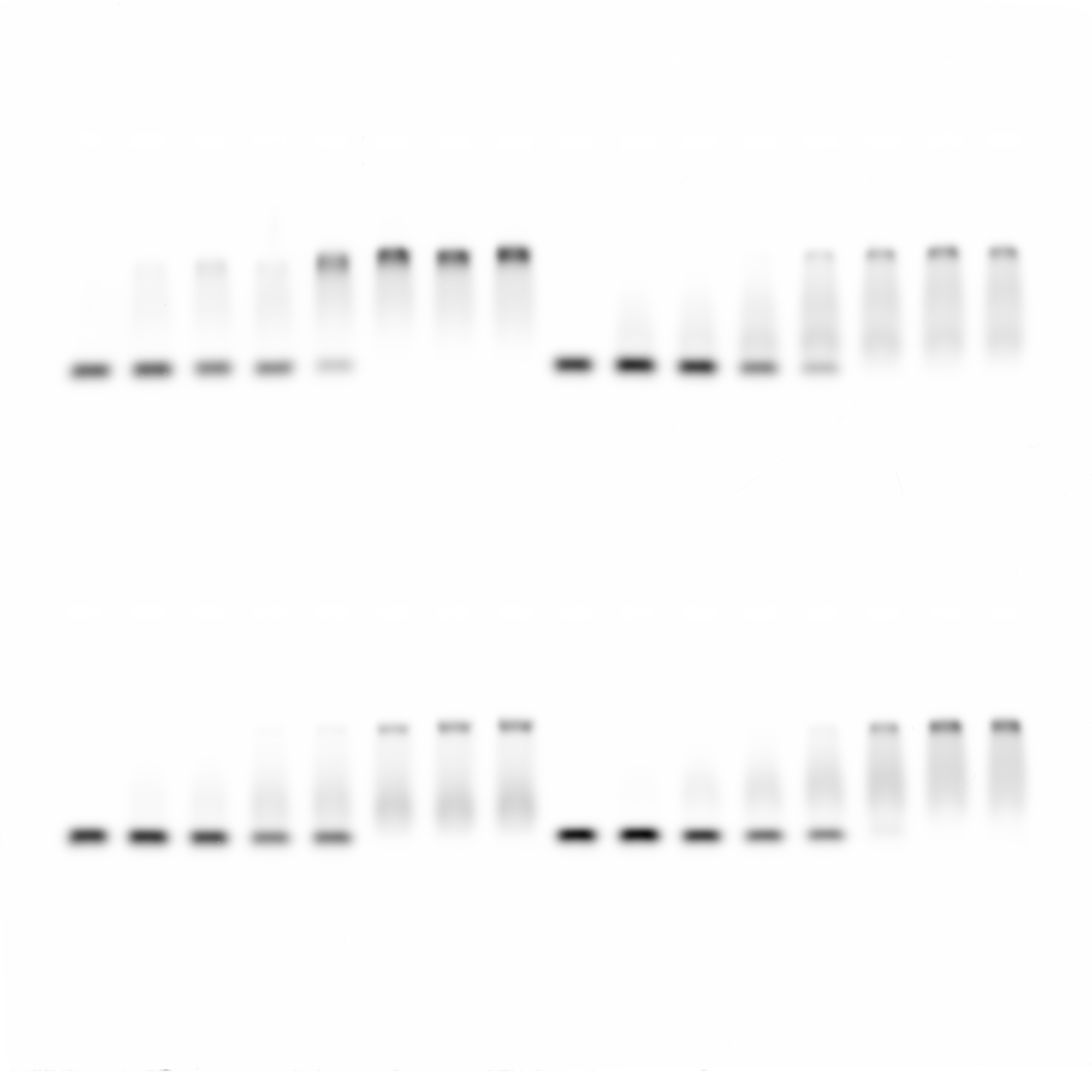

Supplement: Figure 5—figure supplement 1—source data 1. [file elife-107114-fig5-figsupp1-data1.zip › Figure 5-figure supplement 1-source data 1/EMSA ssDNA (F279, 304,306,313) [FAM].tif]

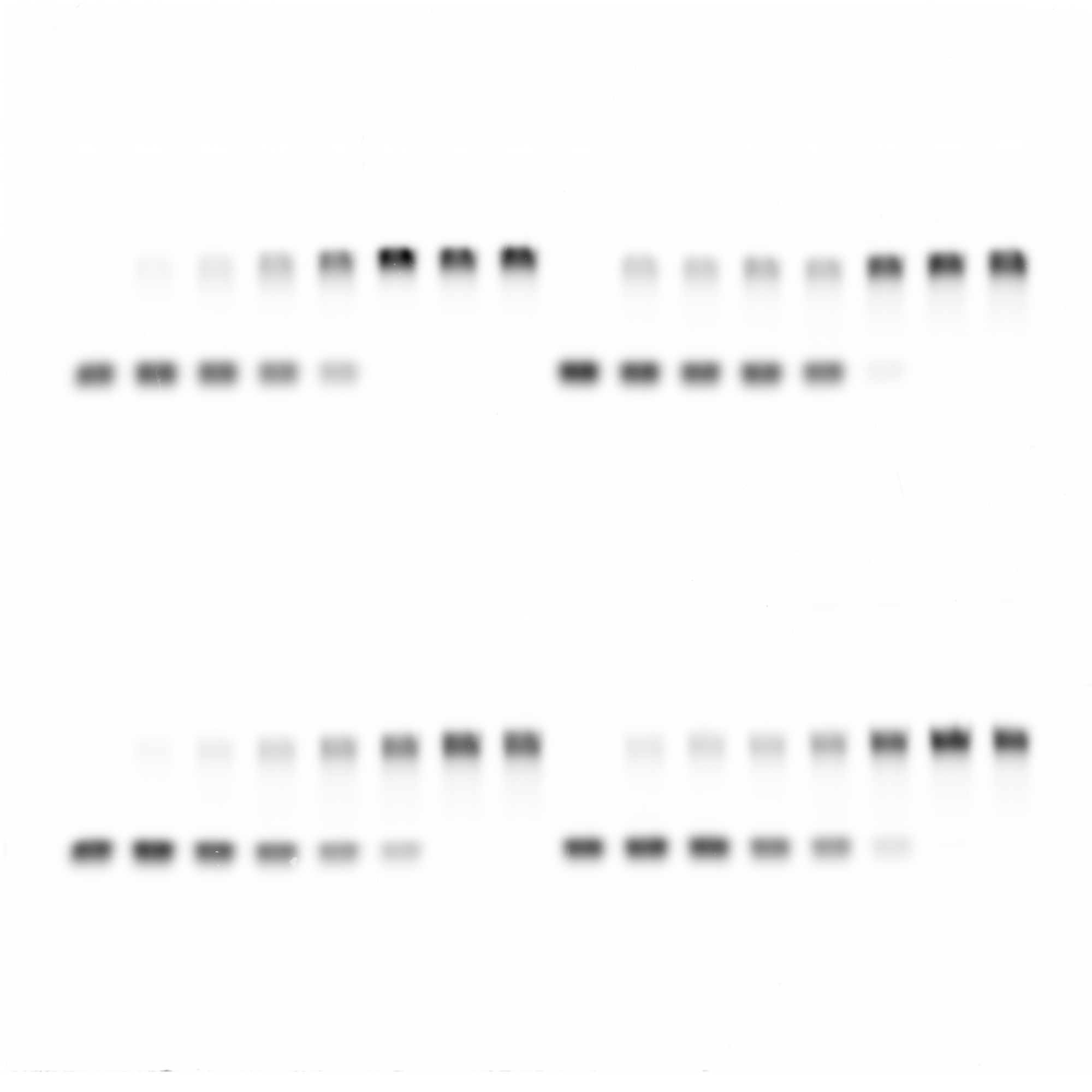

Supplement: Figure 5—figure supplement 1—source data 1. [file elife-107114-fig5-figsupp1-data1.zip › Figure 5-figure supplement 1-source data 1/EMSA dsDNA (WT, 64,39-40,70-73) [FAM].tif]

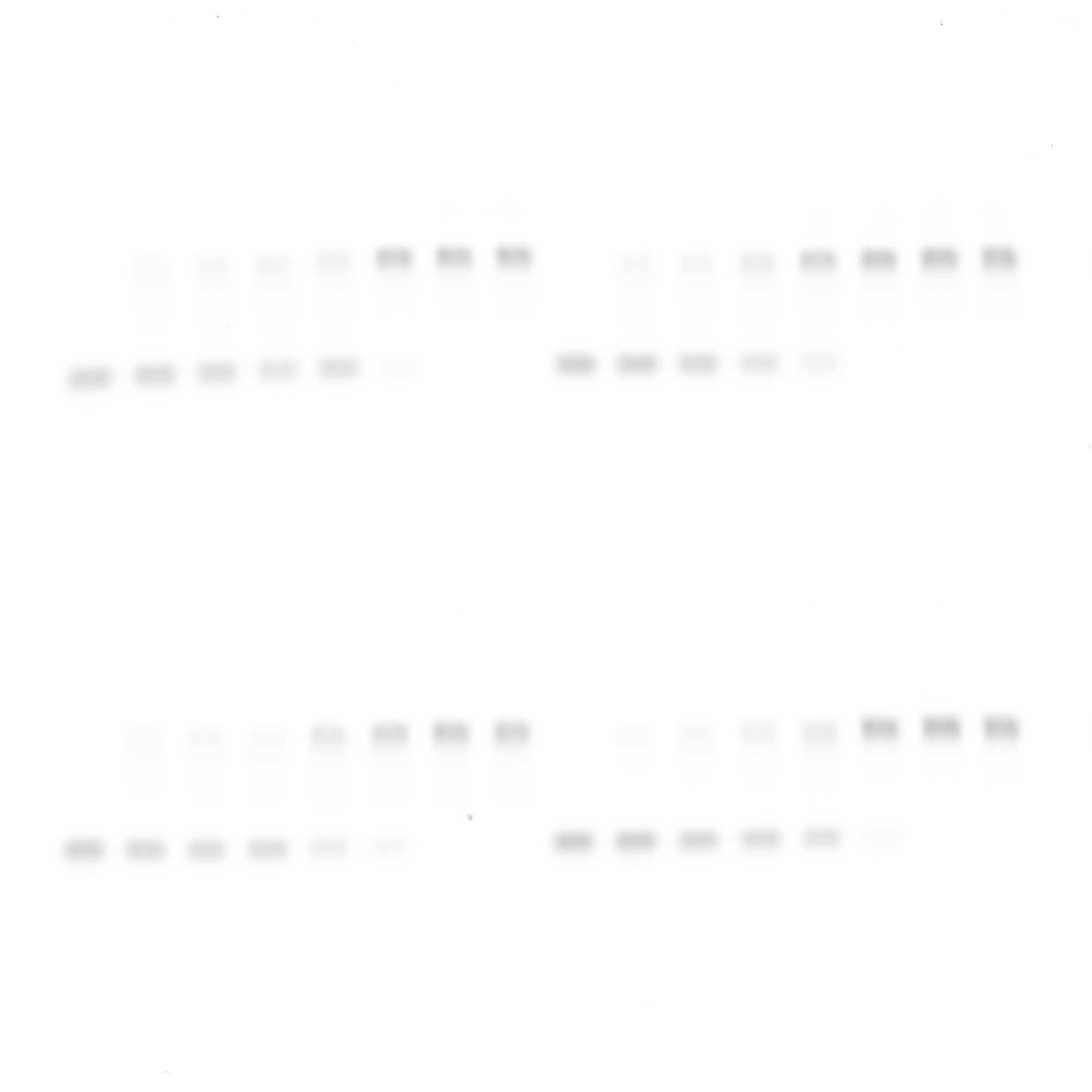

Supplement: Figure 5—figure supplement 1—source data 1. [file elife-107114-fig5-figsupp1-data1.zip › Figure 5-figure supplement 1-source data 1/EMSA dsDNA (279, 304,306,313) [FAM].tif]

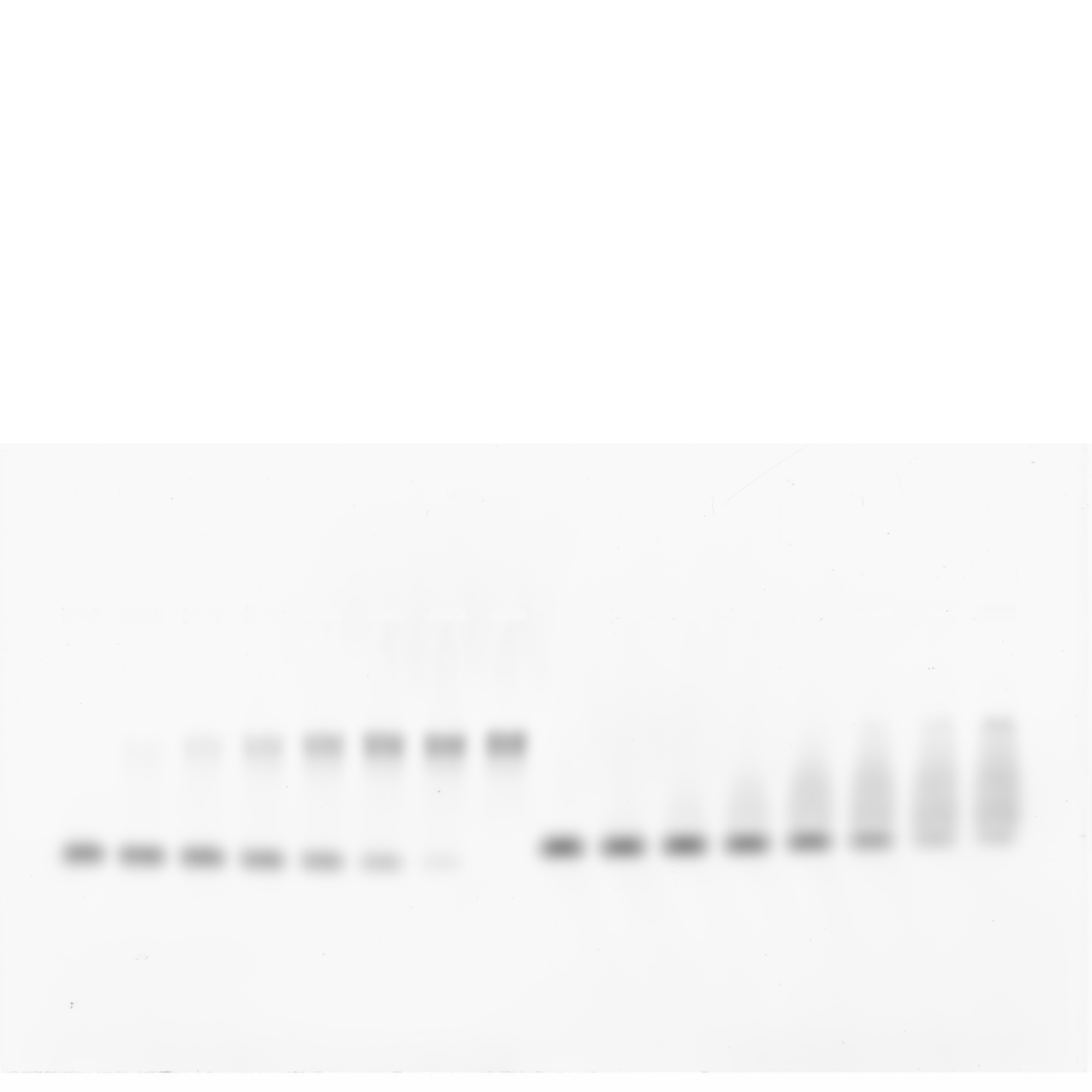

Supplement: Figure 5—figure supplement 1—source data 1. [file elife-107114-fig5-figsupp1-data1.zip › Figure 5-figure supplement 1-source data 1/EMSA ds and ssDNA R303A [FAM].tif]

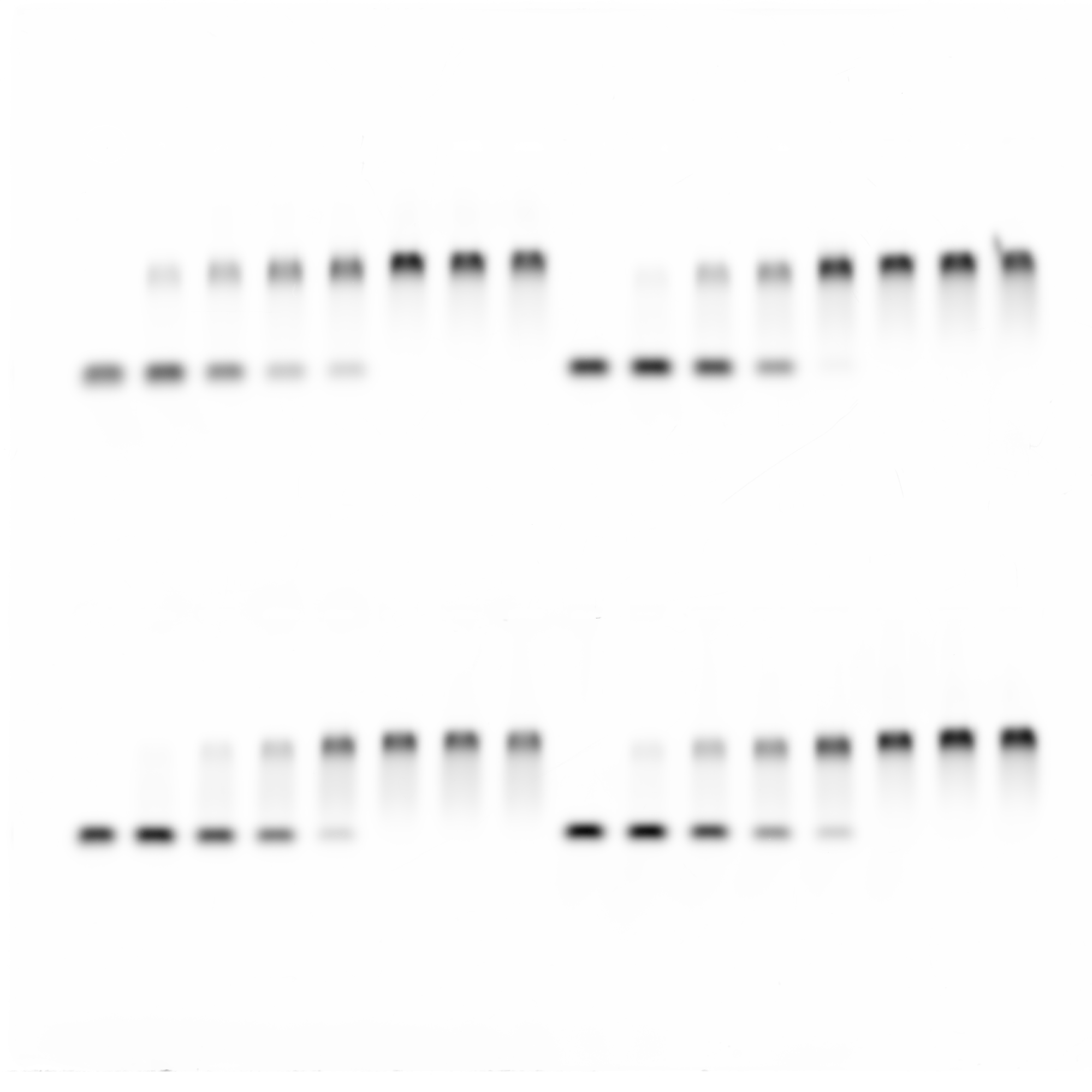

Supplement: Figure 5—figure supplement 1—source data 1. [file elife-107114-fig5-figsupp1-data1.zip › Figure 5-figure supplement 1-source data 1/EMSA ssDNA (WT, 64,39-40,70-73) [FAM].tif]

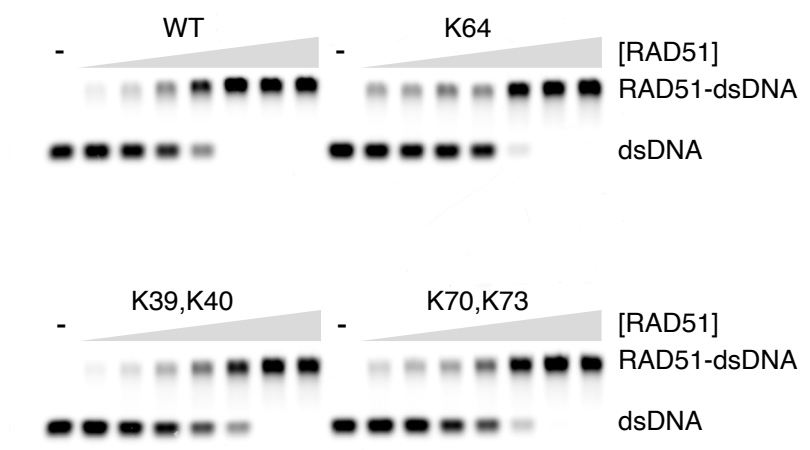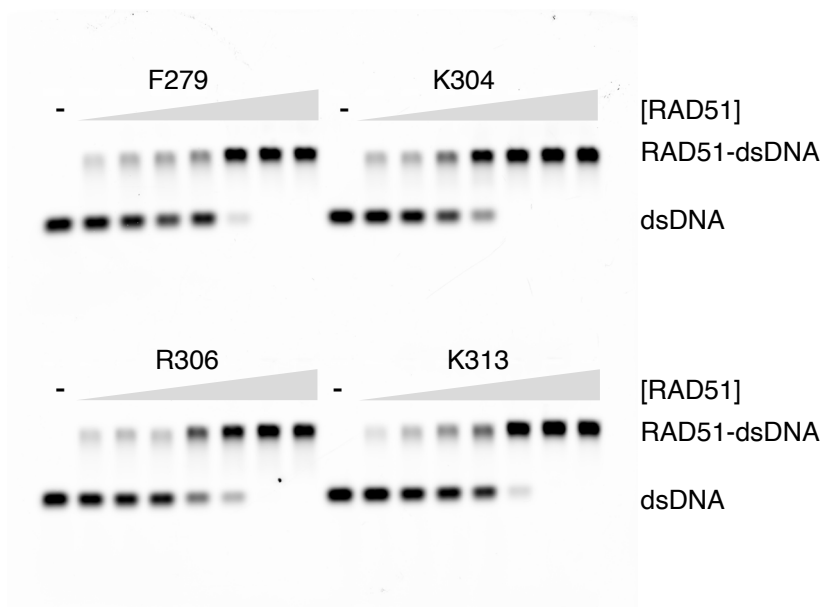

Supplement: Figure 5—figure supplement 1—source data 2. [file elife-107114-fig5-figsupp1-data2.zip › Figure 5-figure supplement 1-source data 2/RAD51mutants_dsDNAbinding.pdf]

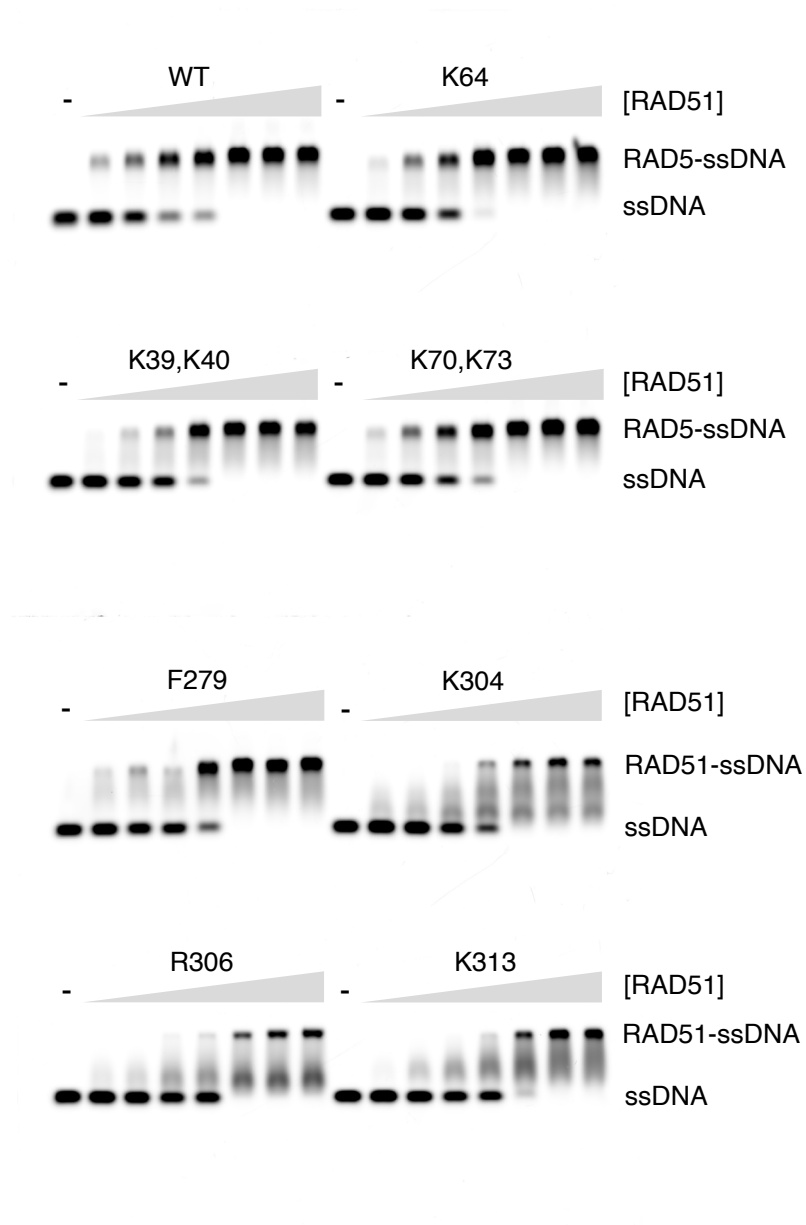

Supplement: Figure 5—figure supplement 1—source data 2. [file elife-107114-fig5-figsupp1-data2.zip › Figure 5-figure supplement 1-source data 2/RAD51mutants_ssDNAbinding.pdf]

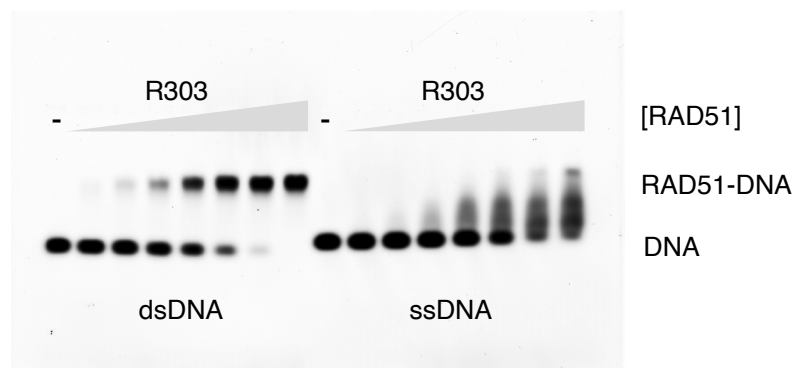

Supplement: Figure 5—figure supplement 1—source data 2. [file elife-107114-fig5-figsupp1-data2.zip › Figure 5-figure supplement 1-source data 2/RAD51-R303A_ss-dsDNAbinding.pdf]
