# Supplementary material for: Structural mechanism of strand exchange by the RAD51 filament
Source: eLife. 2025 Aug 18;14:RP107114. doi: 10.7554/eLife.107114 (PMC12360782; doi:10.7554/eLife.107114)
Supplement: Supplementary file 1. [file elife-107114-supp1.docx]

Oligonucleotide sequence, size and labelling.

|  | **Size (nt)** | **Sequence (5’–3’)** |
| --- | --- | --- |
| *EMSA & Strand Exchange* | | |
| Fig. 5B & Fig. S6B | 60 | ATGGTGTGTGTAGGTTAATGTGAGGAGGAGAGGTGAAGAAGGAGGAGAGAAGAAGGAGGC |
| Fig. 5B | 60 | ATGGTGTGTGTAGGTTAATGTGAGGAGGAGAGGTGAAGAAGGAGGAGAGAAGAAGGAGGC-**FQ** |
| Fig. 5B & Fig. S6A, B | 60 | **FAM**-GCCTCCTTCTTCTCTCCTCCTTCTTCACCTCTCCTCCTCACATTAACCTACACACACCAT |
| Fig. 5B | 60 | TTTTTTTTTTTTTTTTTTTTTTTTTTTTTTTTTTTTTTTTTTTTTTTTTTTTTTTTTTTT |
| Fig. 5D | 49 | **Alexa488**-TCAGGCGTCATTTTTCTGGTACGGAAAGTGATGCGAAAAAAACAGCGGC |
|  | | |
| *D-loop reconstitution and cryoEM* | | |
| Fig. S1B | 50 | TGGAGGTGCATCGAGCTCGCGACAAACCTTCTATGTTGAGCGTCAGTCGG |
| Fig. S1C | 50 | **Biotin**-TGGAGGTGCATCGAGCTCGCGACAAACCTTCTATGTTGAGCGTCAGTCGG |
| Fig. S1B | 50 | CCGACTGACGCTCAACATAGGTACCACACGGCGAGCTCGATGCACCTCCA-**Cy3** |
| Fig. S1C | 50 | **Biotin**-CCGACTGACGCTCAACATAGGTACCACACGGCGAGCTCGATGCACCTCCA-**Cy3** |
| Fig. S1B | 32 | **Cy5**-TTTTTTTTTTTCGTGTGGTACTTTTTTTTTTT |
| Fig. S1C | 32 | **Biotin**-TTTTTTTTTTTCGTGTGGTACTTTTTTTTTTT-**Biotin** |

FAM: 6-carboxyfluorescein; FQ: Iowa Black® (IDT); Cy3: cyanine 3; Cy5: cyanine 5.
