# Supplementary material for: Structural mechanism of strand exchange by the RAD51 filament
Source: eLife. 2025 Aug 18;14:RP107114. doi: 10.7554/eLife.107114 (PMC12360782; doi:10.7554/eLife.107114)
Supplement: Supplementary file 2. [file elife-107114-supp2.docx]

**CryoEM data collection and real-space refinement**

| *Data Collection* | **Dataset #1 (free DNA)** | **Datasest #2 (streptavidin-capped DNA)** |
| --- | --- | --- |
| Microscope | Titan Krios G3 | Titan Krios G3 |
| Voltage (keV) | 300 | 300 |
| Detector | K3 | K3 |
| Collection mode | Counting | Counting |
| Magnification | 130,000x | 130,000x |
| Pixel size (Å) | 0.326 (super-resolution) | 0.326 (super-resolution) |
| Pixel area (Å^2^) | 0.425 | 0.425 |
| Exposure (s) | 1.31 | 1.34 |
| Dose (e^-^/pixel/s) | 15.189 | 15.26 |
| Dose (e^-^/Å^2^/s) | 35.74 | 35.90 |
| Total dose (e^-^/Å^2^) | 46.80 | 48.10 |
| Number of frames/movie | 48 | 50 |
| Dose per frame (e^-^/Å^2^) | 0.975 | 0.96 |
| Num. movies | 2792 | 8960 |
| Defocus range (mm) | -2.6 to -1.0 (0.2) | -2.5 to -0.9 (0.2) |

*Real-space refinement*

| Composition: |  |  | Stereochemistry: |  |  | Model vs data^1^: | Resolution (Å) | |
| --- | --- | --- | --- | --- | --- | --- | --- | --- |
|  |  |  |  |  |  |  | masked | unmasked |
| Chains | 21 |  | Bonds (rmsd): |  |  | FSC, 0.143 | 2.62 | 2.65 |
| Non-H atoms | 24,205 |  | Length(Å) | 0.006 |  | FSC, 0.5 | 2.85 | 3.06 |
| Residues: |  |  | Angles (°) | 0.479 |  | CC, mask | 0.89 | |
| Protein | 2831 |  | MolProbity score^2^ | 1.27 |  | CC, peaks | 0.74 | |
| Nucleotide | 108 |  | Clash score | 5.04 |  | CC, volume | 0.89 | |
| Ligands: |  |  | Ramachandran (%): |  |  |  |  | |
| ATP | 9 |  | Outliers | 0.00 |  |  |  | |
| Ca^2+^ | 18 |  | Allowed | 1.61 |  |  |  | |
|  |  |  | Favoured | 98.39 |  |  |  | |
|  |  |  | Rotamer outliers (%) | 0.49 |  |  |  | |
|  |  |  | <ADP (B-factors)>: |  |  |  |  | |
|  |  |  | Protein | 127.59 |  |  |  | |
|  |  |  | Nucleotide | 210.73 |  |  |  | |
|  |  |  | Ligand | 110.90 |  |  |  | |

1. Afonine PV, Klaholz BP, Moriarty NW, Poon BK, Sobolev OV, Terwilliger TC, Adams PD, Urzhumtsev A. New tools for the analysis and validation of cryo-EM maps and atomic models. Acta Crystallographica Section D: Structural Biology. 2018;74(9):814–840.

2. Chen VB, Arendall WB, Headd JJ, Keedy DA, Immormino RM, Kapral GJ, Murray LW, Richardson JS, Richardson DC. MolProbity: all-atom structure validation for macromolecular crystallography. Acta Crystallographica Section D: Biological Crystallography. 2010;66(Pt 1):12–21.
